# Translation Repression in Human Cells by MicroRNA-Induced Gene Silencing Requires RCK/p54

**DOI:** 10.1371/journal.pbio.0040210

**Published:** 2006-06-13

**Authors:** Chia-ying Chu, Tariq M Rana

**Affiliations:** **1**Department of Biochemistry and Molecular Pharmacology, University of Massachusetts Medical School, Worcester, Massachusetts, United States of America; Oregon State UniversityUnited States of America

## Abstract

RNA interference is triggered by double-stranded RNA that is processed into small interfering RNAs (siRNAs) by Dicer enzyme. Endogenously, RNA interference triggers are created from small noncoding RNAs called microRNAs (miRNAs). RNA-induced silencing complexes (RISC) in human cells can be programmed by exogenously introduced siRNA or endogenously expressed miRNA. siRNA-programmed RISC (siRISC) silences expression by cleaving a perfectly complementary target mRNA, whereas miRNA-induced silencing complexes (miRISC) inhibits translation by binding imperfectly matched sequences in the 3′ UTR of target mRNA. Both RISCs contain Argonaute2 (Ago2), which catalyzes target mRNA cleavage by siRISC and localizes to cytoplasmic mRNA processing bodies (P-bodies). Here, we show that RCK/p54, a DEAD box helicase, interacts with argonaute proteins, Ago1 and Ago2, in affinity-purified active siRISC or miRISC from human cells; directly interacts with Ago1 and Ago2 in vivo, facilitates formation of P-bodies, and is a general repressor of translation. Disrupting P-bodies by depleting Lsm1 did not affect RCK/p54 interactions with argonaute proteins and its function in miRNA-mediated translation repression. Depletion of RCK/p54 disrupted P-bodies and dispersed Ago2 throughout the cytoplasm but did not significantly affect siRNA-mediated RNA functions of RISC. Depleting RCK/p54 released general, miRNA-induced, and *let*-7-mediated translational repression. Therefore, we propose that translation repression is mediated by miRISC via RCK/p54 and its specificity is dictated by the miRNA sequence binding multiple copies of miRISC to complementary 3′ UTR sites in the target mRNA. These studies also suggest that translation suppression by miRISC does not require P-body structures, and location of miRISC to P-bodies is the consequence of translation repression.

## Abstract 2

RCK/p54 plays a critical role in translational repression mediated by miRISC; translation repression by miRISC does not require P-body structures.

## Introduction

Small non-coding RNAs play important roles in the posttranscriptional regulation of genes that code for diverse biological functions, e.g., in most metazoan organisms from nematodes to mammals [ 1– 3]. Two classes of such small (∼ 21 nucleotide [nt]) RNAs that have been extensively studied in gene silencing are short interfering RNAs (siRNAs) and microRNAs (miRNAs) (reviewed in [ 4]). Currently, the best known mechanism of gene silencing is RNA interference (RNAi), an evolutionarily conserved process whereby double-stranded RNA induces the sequence-specific degradation of homologous mRNA [ 5]. Long double-stranded RNA and precursors of miRNAs are processed by Dicer enzyme, and siRNAs are assembled into an RNA-induced silencing complex called RISC. The RNAi machinery can also be programmed in cells by introducing duplexes of siRNAs that are assembled into RNA-induced silencing complexes (siRISC) containing Dicer, argonautes, and other proteins [ 6]. Although RNAi has commonly been associated with siRNAs, this process is largely mediated in plants by miRNAs [ 7, 8], and examples of miRNA-mediated RNAi have been found in mammals and viruses (reviewed in [ 6]). Growing evidence indicates that miRNAs are important in human disease, including cancers [ 9– 12]. For example, relatively low levels of *let*-7 miRNA up-regulate RAS protein in lung cancer cells, demonstrating a possible role of miRNA in tumorigenesis [ 13].

Both classes of small RNAs are assembled into silencing complexes that contain Dicer, argonautes, and other proteins [ 4], but they silence gene expression by two different pathways. Upon recognizing complementary mRNA, activated RISC forms an effector complex with the target mRNA [ 6]. Antisense siRNA in activated RISC serves as a guide for Argonaute2 (Ago2) [ 14– 16] to catalyze the cleavage of target mRNA at a site ∼ 10 nt from the 5′ end of the siRNA [ 17]. Following cleavage, the target mRNA is degraded. Activated RISC, as a multi-turnover enzyme [ 18], is recycled to cleave additional mRNA targets.

In the case of miRNAs, they are assembled into miRNA-induced silencing complexes (miRISC) that contain Dicer, argonaute proteins, transactivation-responsive RNA-binding protein [ 19– 22], and other cellular factors [ 4]. This assembly into miRISC has been implicated in miRNA functions [ 19– 24].

Whether siRNA-mediated RNAi or miRNA-mediated inhibition of translation is triggered depends largely on the degree of complementarity between the siRNA or miRNA and its mRNA target (reviewed in [ 2, 25]). While both miRNAs and siRNAs must harbor sequences that recognize the target mRNA, miRNAs are generally not fully complementary to the mRNA target. In contrast, siRNA sequences must be completely complementary to the mRNA target cleavage site to efficiently induce cleavage through the RNAi pathway. Interestingly, miRNAs can behave like siRNAs and induce mRNA cleavage when the miRNA sequence is completely complementary to a target mRNA [ 18, 26, 27].

In human cells, the mechanism by which the endogenous miRNA and siRNA pathways are distinguished is not clearly understood. It is also unclear how miRNAs repress the translation of target mRNAs. New insights into miRNA function have recently been provided by localization of the RISC components, Ago1 and Ago2, in mRNA-processing bodies (P-bodies) [ 28– 31], which are cytoplasmic foci containing translationally repressed mRNP complexes. During cellular translational control, an mRNP complex is formed containing the translationally repressed mRNA and associated repressor proteins and lacking translation initiation factors [ 32]. These translationally repressed mRNPs accumulate in P-bodies and contain proteins that mediate the translation [ 33– 35], RNAi [ 28– 31, 36], translation suppression [ 37], and decay [ 32, 38] of cellular mRNA. P-bodies, also referred to as GW or Dcp bodies, contain GW182 proteins that have recently been reported to play a role in RNAi [ 39– 41]. Other proteins found in cytoplasmic P-bodies and implicated in mRNA processing are RCK/p54, Lsm1, Dcp1:2, and eIF4E [ 32, 33, 38]. Given that target mRNA and RISC components have been co-localized in P-bodies [ 29, 31], it is possible that P-bodies are the bona fide site for RISC-induced target cleavage or repression of translation. However, the mechanism by which target mRNA and miRISC are directed to P-bodies and how translation is repressed by miRISC is still unknown.

One P-body protein, RCK/p54, the human homolog of yeast Dhh1p, is a member of the ATP-dependent DEAD box helicase family and was originally identified as a proto-oncogene [ 42]. In human cells, RCK/p54 interacts in P-bodies with the translation initiation factor, eIF4E [ 33]. The *Xenopus* homolog of RCK/p54, Xp54, which interacts with eIF4E and forms RNA-dependent oligomers, represses the translation of mRNA in oocytes and eggs [ 43]. In yeast, Dhh1p interacts with the decapping and deadenylase complex and functions in translational repression [ 44]. Dhh1p has also recently been shown to stimulate translational repression by inhibiting production of the pre-initiation complex [ 45].

Here, we show that RCK/p54 interacts with argonaute proteins, Ago1 and Ago2, in affinity-purified active RISC assemblies from human cells programmed with siRNA or endogenous miRNA; directly interacts with Ago1 and Ago2 in vivo, facilitates formation of cytoplasmic P-bodies, and acts as a general repressor of translation. Depletion of RCK/p54 disrupted P-bodies and dispersed Ago2 throughout the cytoplasm. We further show that depletion of RCK/p54 did not significantly affect the RNAi function of RISC, but released general, miRNA-induced and *let*-7-mediated translational repression. Taken together, our results suggest that RCK/p54 is the effector molecule in miRISC that represses translation and that the specificity of this repression is dictated by the sequence of miRNA binding to complementary sites in the 3′ UTR of the target mRNA.

## Results

### Human Argonaute Proteins Interact with RCK/p54, a Component of P-Bodies

**Figure 1:**
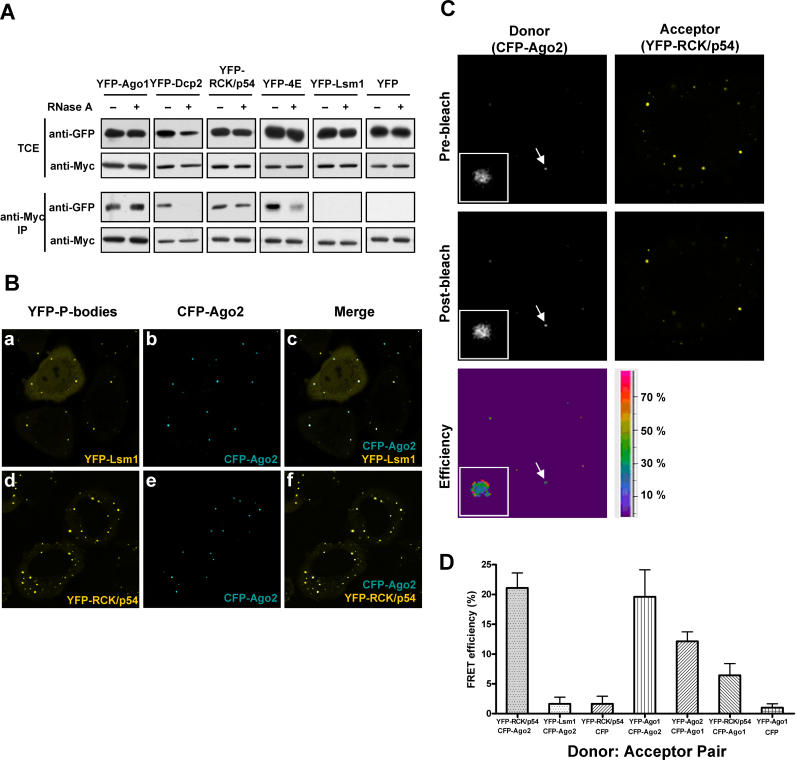
Human Argonaute Proteins Interact with RCK/p54, a Component of P-Bodies (A) Immunoprecipitation and immunoblot analyses. TCEs from HeLa cells co-expressing Myc-Ago2 and YFP-Ago1, YFP-Dcp2, YFP-RCK/p54, YFP-eIF4E, YFP-Lsm1, or YFP were treated with +/− RNase A followed by Myc-Ago2 immunoprecipitation. TCE and anti-Myc IPs were analyzed by immunoblot using anti-GFP and anti-Myc antibodies. (B) In vivo localization of RCK/p54 and Ago2 to P-bodies. HeLa cells expressing YFP-Lsm1 and CFP-Ago2 (a, b, and c), YFP-RCK/p54 and CFP-Ago2 (d, e, and f) were visualized by confocal microscopy at 24 h post-transfection. (C) Visualization of interactions between RCK/p54 and Ago2 in P-bodies by FRET. HeLa cells expressing YFP-RCK/p54 and CFP-Ago2 were fixed at 24 h post-transfection. FRET was measured by an acceptor photobleaching method. Fluorescence images of donor (CFP-Ago2) and acceptor (YFP-RCK/p54) molecules were taken before and after photobleaching YFP. FRET efficiencies were calculated as described [ 48, 49, 68], and data were analyzed by Leica confocal software. Arrows point to P-bodies, which are enlarged in insets. (D) FRET efficiencies between different P-body protein donor: acceptor pairs. HeLa cells co-expressing YFP-RCK/p54 and CFP-Ago2, YFP-Lsm1 and CFP-Ago2, YFP-RCK/p54 and CFP, YFP-Ago1 and CFP-Ago2, YFP-Ago2 and CFP-Ago1, YFP-RCK/p54 and CFP-Ago-1, as well as YFP-Ago1 and CFP, were fixed and FRET efficiencies were measured.

To investigate the mechanism of miRNA-mediated repression of mRNA translation and to determine the interactions of P-body components with the RNAi machinery, we constructed expression vectors for the yellow fluorescent protein (YFP)-tagged P-body proteins, Lsm1, RCK/p54, Dcp2, and eIF4E. These vectors were co-expressed in HeLa cells with Myc-tagged Ago2 and immunopurified using anti-Myc antibodies. The protein composition of isolated complexes was analyzed by immunoblot using antibodies against green fluorescent protein (GFP) or Myc. When total cell extracts (TCE) were analyzed to determine the protein expression efficiencies of the vectors used in these experiments ( Figure 1A, TCE lane), all YFP- and Myc-tagged proteins were expressed. Analysis of immunopurified complexes revealed that Ago1, Dcp2, RCK/p54, and eIF4E formed complexes with Ago2 ( Figure 1A, anti-Myc lane). Control experiments showed that YFP did not co-purify with Ago2 ( Figure 1A). Interestingly, Lsm1 did not co-purify with Ago2, but localized to P-body structures in HeLa cells ( Figure 1B).

Since P-bodies contain RNA and proteins, many protein components of P-bodies are likely to be assembled on a common RNA scaffold without forming functional protein–protein interactions. To address this possibility, HeLa cells were transfected with vectors to co-express Myc-Ago2 and the YFP-tagged P-body proteins, Lsm1, RCK/p54, Dcp2, and eIF4E, subjected to RNase A digestion, and immunopurified. Analysis of immunopurified complexes showed that Ago1 and RCK/p54 interactions with Myc-Ago2 were not affected by RNase treatment, whereas the amounts of Dcp2 and eIF4E protein that co-purified with Myc-Ago2 decreased significantly ( Figure 1A, anti-Myc lane). Control experiments analyzing Myc-Ago2 showed that equal amounts of complexes were purified ( Figure 1A, anti-Myc lane). Taken together, these results suggest that Ago1 and RCK/p54 directly interacted with Ago2, whereas Dcp2 and eIF4E interactions with Ago2 were RNA-mediated. These results do not rule out the possibility that Ago1 and RCK/p54 interactions with Ago2 were mediated by adapter protein(s).

Cytoplasmic P-bodies contain RCK/p54, Lsm1, Dcp2, and eIF4E [ 32, 33, 38]. To confirm whether these structures also contain Ago2 as recently reported [ 28, 31], we transfected HeLa cells with expression vectors containing YFP-Ago1, CFP (cyan fluorescent protein)-Ago1, YFP-Ago2, and CFP-Ago2. Transiently expressed YFP- and CFP-tagged Ago1 and Ago2 co-localized at specific foci in cytoplasm (unpublished data). To examine the contents of these cytoplasmic foci, HeLa cells were transfected with expression vectors for YFP-Lsm1 and CFP-Ago2, or YFP-RCK/p54 and CFP-Ago2, and visualized 24 h later by confocal microscopy. As shown in Figure 1B, CFP-Ago2 co-localized with YFP-Lsm1 and YFP-RCK/p54, two bona fide P-body proteins [ 32, 33, 38]. In addition, immunofluorescence experiments showed that endogenous Ago2 co-localized with Lsm1 in P-bodies ( Figure S1). Interestingly, over-expressing YFP-RCK/p54 increased the average number of P-bodies in every cell ( Figure 1B, compare a and d). To confirm that the localization of Ago2 and Lsm1 to P-bodies was not the result of over-expression in transiently transfected cells, we immunostained cells with antibodies to endogenous Ago2 and Lsm1 and found that the localization of Ago2 and Lsm1 to P-bodies ( Figure S1) was similar to that in Figure 1B. These results show that Ago2, Lsm1, and RCK/p54 are present in P-bodies.

To visualize protein–protein interactions in vivo, we used fluorescence resonance energy transfer (FRET) as a probe. In FRET, a fluorescent donor molecule transfers energy via a nonradiative dipole–dipole interaction to an acceptor molecule [ 46]. We used a well-known donor: acceptor fluorescent-protein pair, CFP:YFP, with a Förster distance (R _0_) of 4.9 nm [ 47]. To determine whether Ago1 and Ago2 interacted in vivo with each other and with RCK/p54, we measured the FRET efficiency between the donor, CFP-Ago2, and acceptor, YFP-RCK/p54. To do so, we used a method in which the donor signal lost during FRET is restored by deliberately photobleaching the acceptor fluorophore to abolish its capacity as an energy acceptor [ 48– 50]. In cells expressing YFP-RCK/p54 and CFP-Ago2, the FRET efficiency was 21.07% ± 2.52% ( Figure 1C and 1D). In cells expressing CFP-Ago2 and YFP-Lsm1, FRET efficiency was not significant (1.62% ± 1.11%), corroborating our immunoprecipitation results ( Figure 1A). Furthermore, cells co-expressing YFP-RCK/p54 and CFP showed no significant FRET efficiency (1.64% ± 1.28%). Similar to the YFP-RCK/p54 and CFP-Ago2 pair, YFP-Ago1 and CFP-Ago2 showed an efficient FRET (19.61% ± 4.51%), indicating a direct interaction between Ago1 and Ago2 in vivo [ 28].

Interestingly, the FRET efficiency between Ago1 and Ago2 decreased to 12.13% ± 1.6% when we used CFP-Ago1 and YFP-Ago2, indicating that the energy transfer efficiencies were sensitive to the orientation of donor: acceptor pair in the ribonucleoprotein (RNP) complex. Moreover, only moderate energy transfer efficiency (6.41% ± 1.96%) was seen when YFP-RCK/p54 and CFP-Ago1 were used in FRET experiments, suggesting that this donor: acceptor pair was not as ideally oriented for an efficient energy transfer as the pair CFP-Ago1 and YFP-Ago2. Alternatively, RCK/p54-Ago1-Ago2 is assembled in an RNP complex where the donor: acceptor pair is affected by the location of the probe. Nonetheless, the efficiency of energy transfer was well above the background control (0.99%). As a control experiment, cells co-expressing YFP-Ago1 and CFP showed no significant FRET efficiency (0.99% ± 0.67%). Taken together, these results indicate that Ago1 and Ago2 directly interact in vivo with each other and with RCK/p54.

### RCK/p54 Is a Component of RISC Containing the Guide Strand of siRNA

**Figure 2:**
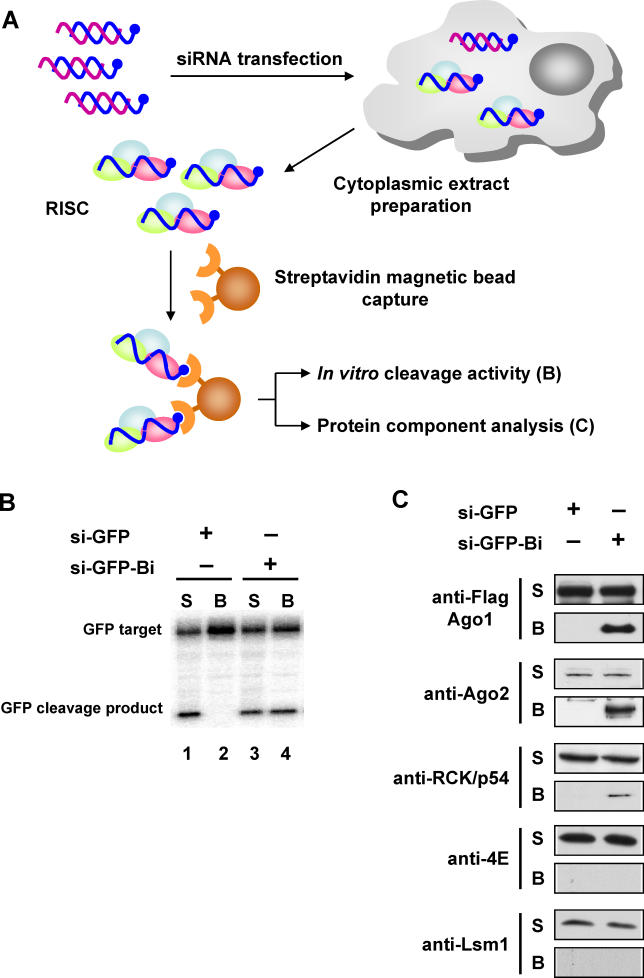
Isolation of Active Human RISC Containing RCK/p54-Ago1/Ago2 (A) Experimental outline to purify active human RISC. The guide strands of siRNA complexes targeting GFP (si-GFP) were conjugated with 3′ biotin (si-GFP-Bi; blue strands) and transfected into HeLa cells. RISCs were captured by incubating cell extracts with streptavidin-magnetic beads. (B) Target mRNA is cleaved by biotin-captured RISC. Bead (B) and supernatant (S) phases of captured RISC were incubated with 124-nt ^32^P-cap-labeled GFP target mRNA. The reactions were stopped after 120 min, and products were resolved on 6% denaturing polyacrylamide gels. (C) Biotin-captured RISC contains proteins associated with mRNA processing. Active human RISC from HeLa cells expressing Flag-Ago1 was captured by biotin-siRNA and its protein composition was analyzed by immunoblot using anti-Flag, anti-Ago2, anti-RCK/p54, anti-Lsm1, and anti-eIF4E antibodies.

To determine whether RCK/p54 is recruited into a functional RISC complex containing argonaute proteins or its association with Ago1/Ago2 is merely due to their co-localization in P-bodies, we affinity-purified active RISCs, analyzed their protein composition, and assayed for RISC function ( Figure 2A). siRNA duplexes targeting GFP (siGFP) were synthesized and 3′ -biotin moieties were conjugated to the 3′ -end of the guide strand. These two duplexes (unmodified siGFP and biotin-modified siGFP) were transfected into HeLa cells, and RISCs were captured by incubating cell extracts with streptavidin-conjugated magnetic beads [ 51, 52]. Beads and supernatants were analyzed for RISC function, i.e., the ability to cleave target mRNA in vitro [ 53]. RISCs primed with siGFP or biotin-modified siGFP guide strands efficiently cleaved their target mRNA ( Figure 2B, lanes 1 and 3), but only biotin-containing RISC purified on streptavidin-magnetic beads showed cleavage activity ( Figure 2B, lanes 2 and 4). These results show that active RISC can be programmed in human cells by biotin-containing guide strands of siRNAs and can be captured on magnetic beads for further analysis.

To probe the involvement of P-body proteins in this purified active RISC, its protein composition was analyzed by immunoblot using antibodies against Flag tag or endogenous Ago2, RCK/p54, eIF4E, and Lsm1. When the RNAi machinery was primed with biotin-containing siRNAs, Ago1, Ago2, and RCK/p54 were co-purified with RISC; and when the guide strand of siRNA did not contain biotin, RISC did not bind to beads ( Figure 2C). Interestingly, eIF4E did not co-purify with RISC, indicating that the Myc-Ago2 interaction with eIF4E that occurred in P-bodies ( Figure 1A) was RNA-dependent, and eIF4E did not directly interact with Ago2 to assemble into active RISC by the guide strand. The data also show that Lsm1 was not a RISC component, consistent with findings shown in Figure 1A. Together, these results identify a new argonaute-interacting protein, RCK/p54, that is recruited to siRISC.

### RCK/p54 Is a Component of RISC Containing miRNA

**Figure 3:**
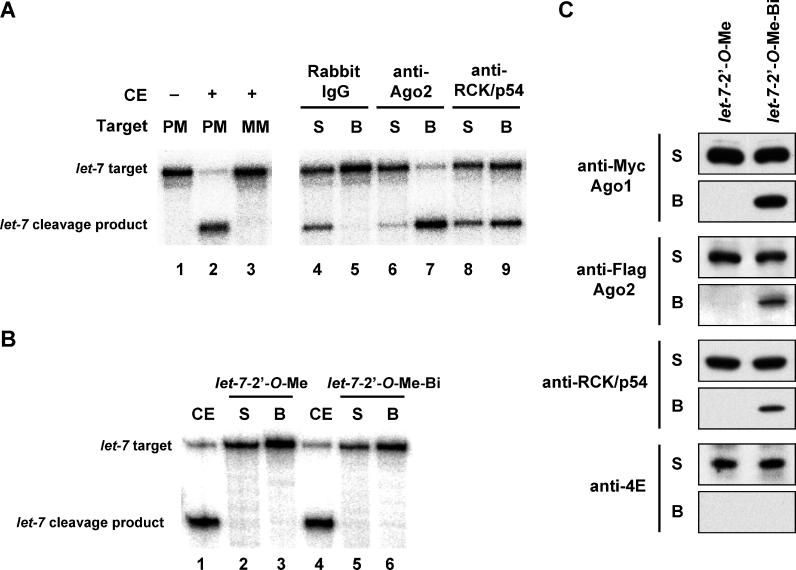
RCK/p54 Is a Component of Human miRISC (A) Affinity-purified miRISCs associated with PCK/p54 retain cleavage activity. To purify miRISC associated with RCK/p54, magnetic protein A beads coupled with rabbit IgG, rabbit anti-Ago2, or rabbit anti-RCK/p54 antibodies were incubated with HeLa cytoplasmic extracts. After immunoprecipitation, RISC activities were analyzed by incubating the supernatant (S) or bead (B) phases with 182-nt ^32^P-cap-labeled *let*-7 substrate mRNAs having a perfectly complementary or mismatched sequence to the *let-*7 miRNA. Cleavage products were resolved on 6% denaturing polyacrylamide gels. CE, cytoplasmic extract; PM, perfect match; MM, mismatch. (B) Affinity-purified miRISCs retain cleavage activity. *let*-7 miRISC cleavage of a perfectly matched RNA target was inhibited by 2′- *O*-Me oligonucleotides complementary to *let*-7 miRNA ( *let*-7–2′- *O-*Me or *let*-7–2′- *O-*Me-biotin). A 182-nt ^32^P-cap-labeled *let*-7 substrate mRNA was incubated with the supernatant (S) or bead (B) phases of captured miRISC. The reactions were stopped after 120 min, and products were resolved on 6% denaturing polyacrylamide gels. (C) miRISCs contain proteins associated with mRNA processing. Cytoplasmic extracts of HeLa cells expressing Flag-Ago2 and Myc-Ago1 were incubated with 2′- *O*-Me oligonucleotides complementary to *let*-7 miRNA ( *let*-7–2′- *O-*Me or *let*-7–2′- *O-*Me-biotin), affinity-purified by streptavidin-magnetic beads to capture *let*-7 miRISC. Supernatant (S) and beads (B) after biotin capture were analyzed by immunoblot using anti-Myc, anti-Flag, anti-RCK/p54, and anti-eIF4E antibodies.

To determine the functional interactions of RCK/p54 with miRISC, we employed affinity purification of RISC and target mRNA cleavage capabilities of miRISC when the target has perfectly complementary sequences to the miRNAs. Cell extracts containing *let-*7 miRISC cleaved perfectly matched radiolabeled target mRNA with high efficiencies, whereas a substrate mRNA containing a mismatched sequence was not cleaved ( Figure 3A, lanes 2 and 3). In the absence of extracts, no mRNA cleavage or degradation was detected ( Figure 3A, lane 1). After establishing the functional assay to analyze miRISC, we next affinity-purified miRISC on magnetic beads using anti-Ago2 and anti-RCK/p54 antibodies and assessed the cleavage of perfectly matched *let*-7 target mRNA by the bead and supernatant phases. miRISCs purified by anti-Ago2 and anti-RCK/p54 antibodies showed efficient cleavage of *let-*7 target mRNA ( Figure 3A, lanes 7 and 9). Ago2 antibodies captured most of the RISC activity on beads as compared to RCK/p54 antibodies ( Figure 3A, lanes 6–9). Non-specific IgG did not purify miRISC activities on beads ( Figure 3A, lanes 4 and 5). These results demonstrate that RCK/p54 is a component of functional miRISC.

To confirm these results, we next used an alternative approach to determine whether RCK/p54 is associated with endogenous RISC programmed by miRISC. RISCs containing miRNA were isolated from HeLa cells by affinity-capture as described in Figure 2A, with the following modifications. To specifically capture endogenous miRISC, 2′- *O-*methyl (2′- *O* Me) inhibitors of *let*-7 miRNA were employed [ 54, 55]. HeLa cytoplasmic extracts expressing Myc-Ago1 and Flag-Ago2 were incubated with 3′-biotinylated- *let*-7–2′- *O*-Me inhibitor, which is complementary to the *let*-7 miRNA, and incubated with streptavidin-conjugated magnetic beads. As a control, cell extracts were treated with the *let-*7–2′- *O*-Me inhibitor without 3′-biotinylation. After capture, beads containing miRISCs were washed with lysis buffer and split into two aliquots to determine miRISC function and protein composition. Function was determined by assaying for in vitro cleavage of a ^32^P-target mRNA that perfectly matched the *let*-7 sequence. The data in Figure 3B (lanes 1 and 4) show that cell extracts containing active *let*-7 miRISC cleaved the perfectly matched target mRNA with high efficiencies [ 54, 55]. Incubation with *let*-7 inhibitors (with or without 3′-biotin) blocked the cleavage activity of *let*-7 miRISC in supernatant or beads ( Figure 3B, lanes 2–3, and lanes 5–6). These results are consistent with previous reports [ 54, 55] that adding 2′- *O*-Me oligonucleotides complementary to *let*-7 abolishes target cleavage activity by *let*-7 in cell extracts, indicating complete hybridization of the 2′- *O*-Me probe. Immunoblot analysis of affinity-purified miRISC using anti-Myc, anti-Flag, anti-RCK/p54, and anti-eIF4E antibodies ( Figure 3C) showed that Myc-Ago1, Flag-Ago2, and RCK/p54 were associated with *let*-7 miRISC. As observed for the active RISC programmed with siRNA, eIF4E did not associate with miRISC. These findings reveal that RCK/p54 is a novel component of endogenous RISC programmed with either siRNA or miRNA.

### Depletion of RCK/p54 Disrupts P-Bodies and Ago2 Localization

**Figure 4:**
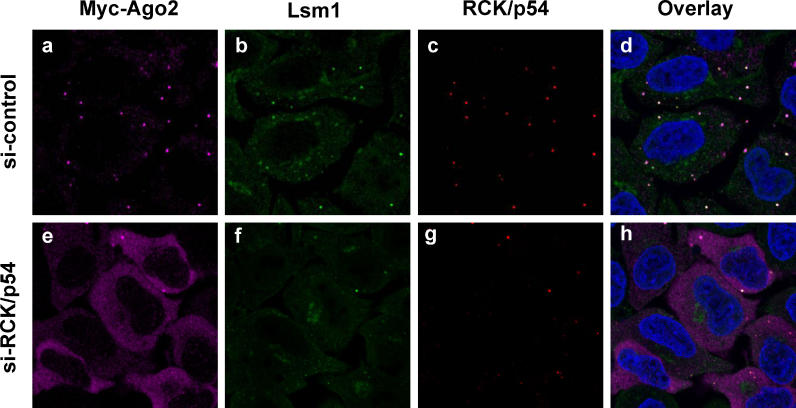
Depletion of RCK/p54 Disrupts P-bodies and Disperses the Localization of Human Ago2 HeLa cells were co-transfected with Myc-Ago2 and siRNA against human RCK/p54 (lower panels) or CDK9 mismatch (control; upper panels). At 24 h post-transfection, cells were analyzed by immunofluorescence using antibodies against Myc-Ago2 (A and E) and against the P-body proteins Lsm1 (B and F) and RCK/p54 (C and G). Cells were stained with Hoechst 33258 to visualize nuclei and images were digitally merged (D and H).

To understand the function and role of RCK/p54 in the RNAi pathway, RCK/p54 was depleted in P-bodies of HeLa cells by siRNA-mediated RNAi. 24 h after transfecting cells with siRNA, real-time quantitative PCR showed that mRNA levels decreased by more than 90% and immunoblot analysis showed that RCK/p54 protein levels decreased significantly without affecting the levels of other P-body proteins including Lsm1 and Ago2 ( Figure S2). The effect of depleting RCK/p54 on localization of Ago2 was next examined by immunofluorescence analysis of HeLa cells expressing Myc-Ago2 and siRNAs against RCK/p54. As shown in Figure 4B, 4C, 4F, and 4G, depleting RCK/p54 disrupted the cellular P-body structures. In these P-body-deficient cells, Ago2 proteins were diffused throughout the cytoplasm and no longer accumulated at specific foci ( Figure 4A and 4E). This cytoplasmic redistribution of Ago2 suggests that its localization to P-bodies is driven in mammalian cells by factors such as RCK/p54.

### Depletion of Lsm1 Disrupts P-Bodies but Does Not Affect RCK/p54 and Ago2 Interactions

**Figure 5:**
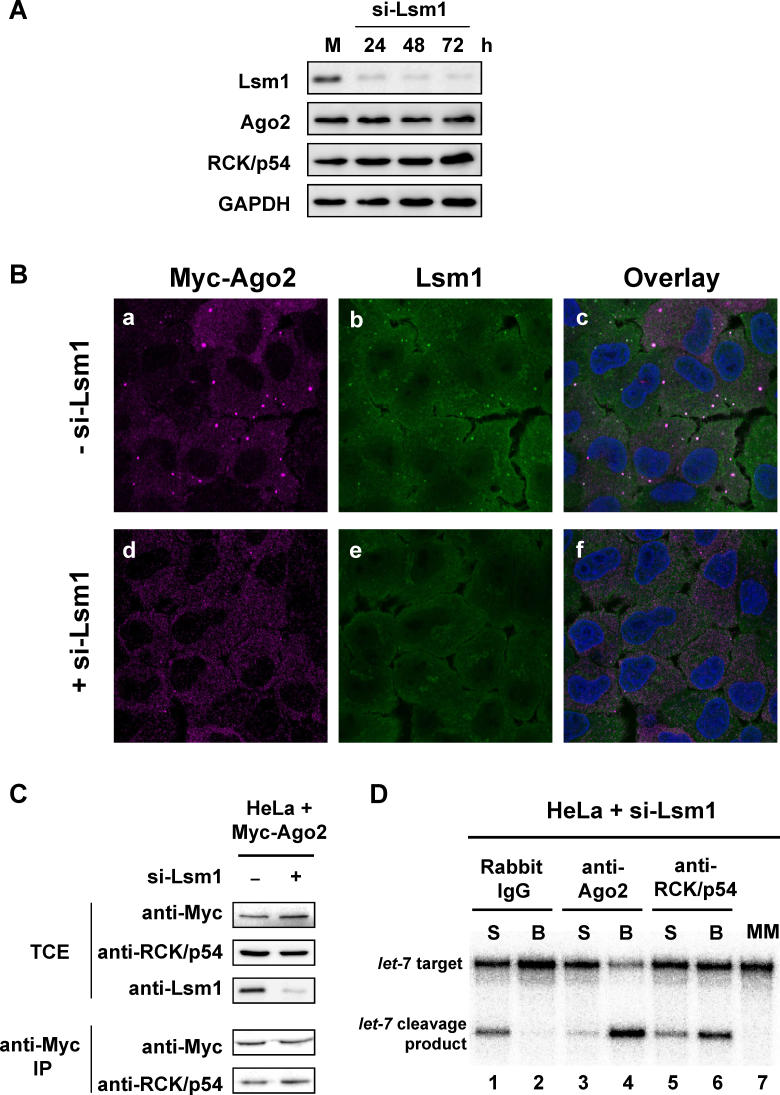
Depletion of Lsm1 Disrupts P-Bodies but Does Not Affect the Interaction between RCK/p54 and Ago2 (A) Specific knockdown of Lsm1 in HeLa cells by siRNA. HeLa cells were transfected with siRNA against Lsm1 and harvested at 24, 48, and 72 h post-transfection, and TCEs were analyzed by immunoblot with antibodies against Lsm1 or GAPDH. (B) Depletion of Lsm1 disrupts P-bodies. HeLa cells were transfected with siRNA against Lsm1. At 48 h post-transfection, cells were analyzed by immunofluorescence using antibodies against Lsm1 and Myc tag for Ago2. Cells were stained with Hoechst33258 to visualize nuclei, and images were digitally merged. (C) RCK/p54 interacts with Myc-Ago2 in Lsm1-depleted cells. HeLa cells were transfected for 48 h with Myc-Ago2 and control siRNA or siRNA against Lsm1, TCEs were prepared, and Myc-Ago2 was immunoprecipitated from an aliquot of TCE. TCE and anti-Myc IPs were analyzed by immunoblot using anti-Myc, anti-RCK/p54, and anti-Lsm1 antibodies. (D) Affinity-purified RCK/p54 and Ago2 from Lsm1-depleted cell extracts retain miRISC activity. HeLa cells were transfected for 48 h with siRNA against Lsm1, and cytoplasmic extracts were prepared. These extracts were incubated with magnetic protein A beads coupled with rabbit IgG, rabbit anti-Ago2, or rabbit anti-RCK/p54 antibodies to purify miRISC associated with RCK/p54. After immunoprecipitation, RISC activities were analyzed by incubating the supernatant (S) or bead (B) phases with 182-nt ^32^P-cap-labeled *let*-7 substrate mRNAs having a perfectly matched or a mismatched sequence to the *let-*7 miRNA. Cleavage products were resolved on 6% denaturing polyacrylamide gels. MM, mismatch.

The interactions between the P-body proteins, RCK/p54, and argonautes raised the question whether P-body structures are required for these interactions. To address this question, we disrupted P-body structures by using RNAi against Lsm1, which is a P-body protein that does not associate with RISC as shown in Figure 1. HeLa cells were transfected with siRNA against Lsm1, harvested at 24, 48, and 72 h post-transfection, and TCEs were analyzed by immunoblot. The results show an efficient knockdown of Lsm1 without changing the levels of RCK/p54, Ago2, and GAPDH ( Figure 5A). We next visualized Lsm1 and Ago2 by immunofluorescence using antibodies against Lsm1 and Myc tag and found that Lsm1 and Ago2 co-localized in P-bodies ( Figure 5B). To determine the status of P-body structures and RISC localization in cells after Lsm1 knockdown, Lsm1-depleted cells were analyzed by immunofluorescence. We found that P-body structures were drastically disrupted, Ago2 was diffused throughout the cytoplasm, and Lsm1 and Ago2 were minimally co-localized ( Figure 5B). These results show that P-body structures were drastically disrupted and Ago2 was diffuse throughout the cytoplasm when Lsm1 was depleted by RNAi.

To determine the effect of P-body disruption on Ago2 and RCK/p54 interactions, we immunopurified Myc-Ago2 and RCK/p54 after Lsm1 knockdown. HeLa cells were transfected for 48 h with Myc-Ago2 and control siRNA or siRNA against Lsm1, TCEs were prepared, and Myc-Ago2 was immunoprecipitated from an aliquot of TCE. TCEs and anti-Myc immunoprecipitation products were analyzed by immunoblot using anti-Myc, anti-RCK/p54, and anti-Lsm1 antibodies. Lsm1 siRNA treatment efficiently depleted Lsm1 protein levels without affecting Myc-Ago2 and RCK/p54 levels ( Figure 5C). Immunoprecipitation using Myc antibodies showed that RCK/p54 interacted with Ago2 and this interaction was not significantly changed by depleting Lsm1 ( Figure 5C). Since P-bodies were disrupted by Lsm1 depletion, these results demonstrate that Ago2 and RCK/p54 interaction does not require P-bodies.

Next, we analyzed the target mRNA cleavage activities of affinity-purified miRISC from Lsm1-depleted cell extracts. P-bodies were disrupted in HeLa cells by transfecting them with siRNA against Lsm1, and cytoplasmic extracts were prepared 48 h post-transfection. These HeLa cell extracts were incubated with magnetic protein A beads coupled with rabbit IgG, rabbit anti-Ago2, or rabbit anti-RCK/p54 antibodies to purify miRISC associated with RCK/p54 or Ago2. After immunoprecipitation, RISC activities were analyzed by incubating the supernatant (S) or bead (B) phases with 182-nt ^32^P-cap-labeled *let*-7 substrate mRNAs having a perfectly complementary or mismatched sequence to the *let-*7 miRNA. Cleavage products were resolved on 6% denaturing polyacrylamide gels. miRISCs purified by anti-Ago2 and anti-RCK/p54 antibodies showed efficient target *let-*7 cleavage ( Figure 5D, lanes 4 and 6). Ago2 antibodies captured more RISC activity on beads than RCK/p54 antibodies, as also observed in Figure 3A (lanes 6–9). Control experiments using IgG ( Figure 5D, lanes 1 and 2) did not show any miRISC activity purified by magnetic beads, supporting specific capture by Ago2 and RCK/p54 antibodies ( Figure 5D, lanes 4 and 6). miRISC did not cleave a mismatched substrate RNA for *let*-7 ( Figure 5D, lane 7). Despite the significant loss of P-body structures in cells treated with Lsm1 siRNA, the efficiency of *let*-7 target mRNA cleavage by miRISC purified by anti-RCK/p54 antibody was not significantly affected (compare Figure 3A, lane 9 with Figure 5D, lane 6). These results indicate that disrupting P-bodies by depleting Lsm1 did not significantly affect RCK/p54 association with miRISC.

### Depletion of RCK/p54 Does Not Affect RNAi Activity In Vivo

To examine whether the P-body protein RCK/p54, also a component of RISC, is involved in siRNA-mediated gene silencing, the siRNA dose dependence of RNAi-mediated gene silencing was quantified in RCK/p54-depleted HeLa cells using a dual fluorescence reporter assay. Briefly, GFP and red fluorescent protein (RFP) were constitutively expressed in cells transfected with reporter plasmids for enhanced GFP (EGFP) and RFP, respectively. GFP expression was silenced by treating cells with a 21-nt siRNA targeting nt 238–258 of the EGFP mRNA. The fluorescence intensity ratio of target (GFP) to control (RFP) fluorophore was determined in the presence of siRNA duplexes and normalized to that observed in control-treated cells [ 52].

**Figure 6:**
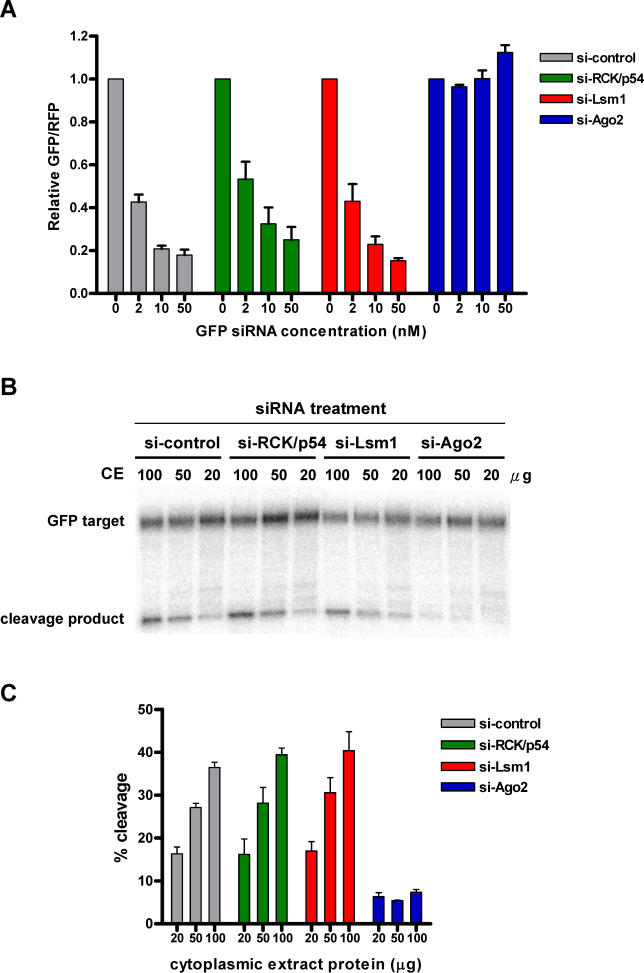
Depletion of RCK/p54 Has No Significant Effect on RNAi Activity In Vivo and In Vitro (A) In vivo effect of depleting RCK/p54, Lsm1, and Ago2 on siRNA dose-dependent RNAi activity. HeLa cells were transfected with siRNAs against CDK9 mismatch (control), RCK/p54, Lsm1, or Ago2. 24 h later cells were transfected again with EGFP and RFP reporter plasmids and varying amounts (2, 10, and 50 nM) of siRNA against EGFP. 24 h after the second transfection, RNAi efficiencies were analyzed (see Materials and Methods). To quantify the effect of depleting RCK/p54 and Ago2 on RNAi, the ratio of GFP/RFP signals was normalized to that observed in the absence of GFP siRNA (0 nM). Normalized ratios < 1.0 indicate specific RNAi at a given siRNA concentration. (B) Depletion of RCK/p54 has no effect on in vitro siRISC cleavage activity. HeLa cells were transfected with siRNAs targeting RCK/p54, Lsm1, Ago2, or CDK9 mismatch (control). 24 h after the first transfection, cells were again transfected with 50 nM siRNA targeting EGFP. 24 h later cytoplasmic extracts were made (see Materials and Methods), and varying amounts (20, 50, 100 μg) of total cytoplasmic extract protein were incubated with a 124-nt, ^32^P-cap-labeled GFP target mRNA. The reactions were stopped after 60 min, and products were resolved on 6% denaturing polyacrylamide gels. (C) Quantification of siRISC cleavage activity in vitro after depletion of RCK/p54, Lsm1, or Ago2. Cleavage activity of siRISC targeting EGFP mRNA was quantified as a function of protein content in extracts of HeLa cells depleted of RCK/p54, Lsm1, or Ago2.

When HeLa cells were transfected with siRNA mismatched for CDK9 (control), the second transfection with GFP siRNA silenced GFP expression in a dose-dependent manner, i.e., GFP/RFP ratios decreased with increasing concentrations of siRNA ( Figure 6A, gray bars). Depleting RCK/p54 before quantifying GFP silencing resulted in moderate to no significant loss of RNAi activity, i.e., GFP/RFP again decreased with increasing concentrations of siRNA ( Figure 6A, green bars). Similarly, depleting Lsm1 ( Figure 6A, red bars) and the decapping enzyme, Dcp2 (unpublished data), before quantifying GFP silencing in HeLa cells did not significantly decrease RNAi activity. In contrast, prior depletion of Ago2 completely abolished RNAi activity ( Figure 6A, blue bars), as expected and consistent with previous reports [ 14– 16]. These results suggest that depleting RCK/p54, which disrupts P-bodies ( Figure 4), did not significantly affect siRNA-mediated RNAi in vivo.

### Depletion of RCK/p54 Does Not Affect RISC-Mediated mRNA Cleavage In Vitro

To examine the role of RCK/p54 in RISC catalysis of mRNA processing, in vitro mRNA cleavage activity was assayed in extracts from RCK/p54-depleted HeLa cells. Cells were transfected with siRNAs against CDK9 mismatch (control), RCK/p54, Lsm1, or Ago2 and programmed 24 h later with GFP siRNA. Varying amounts of cytoplasmic extract (20–100 μg) were assayed in vitro for target mRNA cleavage activity. As shown in Figure 6B and 6C, cleavage activity increased with increasing protein concentration of extracts from both control and RCK/p54-depleted cells, reaching a robust cleavage (37%–41%) of target mRNA at the highest protein concentration (100 μg). In consistent with in vivo results shown in Figure 6A, Ago2 depletion abolished the target mRNA cleavage activities of RISC. These results show that RCK/p54 does not affect the slicer function of RISC in cleaving target mRNA in vitro *.*

### RCK/p54 Represses General and miRNA-Mediated Translation

**Figure 7:**
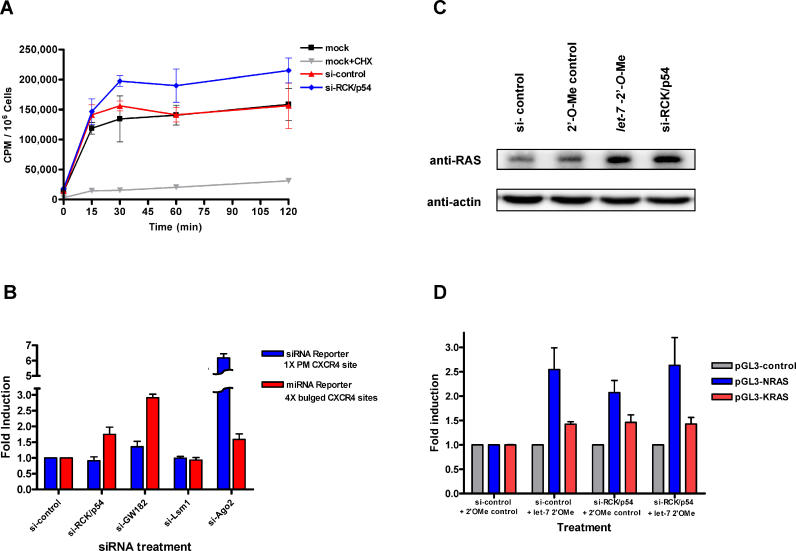
RCK/p54 Represses General and miRNA-Mediated Translation (A) Release of general translational repression by silencing RCK/p54. Incorporation of [ ^35^S]methionine into HeLa cells was used to measure general translational activity. Cells were transfected with 50 nM siRNAs targeting mismatched CDK9 (control) or RCK/p54. Mock control cells were treated with the transfection reagent only. At 24 h post-transfection, cells were incubated for 1 h in medium lacking Met and Cys, and metabolically labeled with [ ^35^S]methionine (see Materials and Methods). As a control for passive uptake of [ ^35^S], mock cells were treated with 40 μg/ml of the translation inhibitor, cycloheximide. Cell incorporation of [ ^35^S] is shown as cpm/10 ^6^ cells versus time after adding [ ^35^S]. (B) Depletion of RCK/p54 releases translational repression in a luciferase reporter system. HeLa cells were transfected with siRNAs against CDK9 mm (control), RCK/p54, GW182, Lsm1, or Ago2. 24 h later, cells were co-transfected with siRNA (1 × perfectly matched [PM] site) or miRNA (4 × bulged sites) luciferase reporters in the presence of CXCR4 siRNA. RL activities were measured 24 h after the second transfection and normalized to FL activity as a control. Repression of reporter gene expression by siRNA or miRNA were measured by normalizing the RL/FL signals in the presence of CXCR4 siRNA to the RL/FL activities observed in the absence of siRNA. Release of gene repression by siRNA or miRNA is presented as the fold induction of RL/FL activities compared to the RL/FL signals observed in control (si-CDK9mm) experiments. FL, firefly luciferase. (C) Depletion of RCK/p54 releases repression of RAS protein translation. HeLa cells were transfected with 100 nM of 2′- *O*-Me oligonucleotide ( *let*-7 2′- *O-*Me inhibitor or 2′- *O-*Me control), and siRNA against RCK/p54 or CDK9 mm control. 24 h after transfection, the cells were counted and harvested (see Materials and Methods). The protein contents of TCEs were resolved by SDS-PAGE and analyzed by immunoblot using anti-RAS and anti-actin antibodies. (D) Depletion of RCK/p54 releases repression of luciferase protein translation by 3′ UTRs of NRAS and KRAS. HeLa cells transfected with an *Rr*-luc-expressing vector, pRL-TK, and a *Pp*- luc-expressing vector, pGL3-control, pGL3-NRAS, or pGL3-KRAS, were co-transfected with 100 nM of 2′- *O-*Me oligonucleotide ( *let*-7 2′- *O-*Me inhibitor or 2′- *O-*Me control) and siRNA against RCK/p54 or CDK9 mm control. TCEs were prepared 48 h post-transfection and dual luciferase assays were performed. Relative GL ( *Pp*-luc)/RL ( *Rr*-luc) signals were normalized to those of pGL3-control-transfected cells and are shown as the fold induction compared to mock treatment.

Our results thus far demonstrate that RCK/p54 is a component of human RISC ( Figures 2 and 3) and enhances P-body formation ( Figure 1). Our data also show that siRNA-mediated RNAi may not require RCK/p54 and P-body cytoplasmic structures. Since we also identified that RCK/p54 associates with miRISC, we hypothesized that RCK/p54 might function in miRNA-mediated suppression of translation. A recent and intriguing report [ 45] identified a yeast homolog of RCK/p54, Dhh1p, which activates decapping and facilitates P-body formation, as a general translation repressor. We therefore reasoned that RCK/p54 might be a general translation repressor used by the miRNA machinery to dictate translation suppression of target mRNAs. To test this hypothesis, we first determined whether RCK/p54 was a general translation repressor in human cells. To this end, RCK/p54 expression was silenced in HeLa cells and general translational activity was analyzed by [ ^35^S]methionine incorporation ( Figure 7A). The translational rates measured by [ ^35^S]-labeling were significantly greater in RCK/p54-depleted cells than in control siRNA-treated cells ( Figure 7A), indicating that RCK/p54 was a translational repressor in human cells, consistent with results in yeast and reticulocyte extracts [ 45].

Since RCK/p54 is a general translational repressor and a component of human RISC, we examined the effects of depleting RCK/p54 and disrupting P-bodies on siRNA- and miRNA-mediated gene silencing. To analyze siRNA- or miRNA-mediated gene-silencing events, we chose a well-established CXCR4 mRNA reporter system [ 56], in which siRNA- or miRNA-reporter constructs harbor 1 × perfectly matched or 4 × bulged CXCR4 siRNA target sites, respectively, in the 3′ UTR of Renilla reniformis luciferase (RL) mRNA [ 56]. In this system, perfectly matched sequences are cleaved by siRISC and bulge-containing sequences are targets for translation suppression by miRISC **.** HeLa cells were co-transfected with siRNAs directed against P-body proteins (RCK/p54, GW182, Lsm1, and Ago2) and with siRNA or miRNA reporters in the absence or presence of 25 nM CXCR4 siRNA. At 24 h post-transfection, cells were harvested and RL activities were analyzed. RL signals were normalized to Photinus pyralis luciferase (FL) signals from cells co-transfected with pGL3 plasmid as control. Depletion of RCK/p54 released only miRNA-mediated gene suppression and had no effect on siRNA-mediated gene silencing ( Figure 7B). Depletion of GW182, an argonaute-interacting P-body protein, released gene suppression by miRNA, consistent with previous findings [ 39– 41], and moderately released siRNA-mediated gene silencing [ 40, 41]. Ago2 depletion significantly released siRNA-mediated gene silencing and moderately released miRNA-mediated gene suppression. Knockdown of Lsm1 had no significant effect on either miRNA- or siRNA-mediated gene silencing. Taken together, these results show that RCK/p54 depletion releases miRNA-mediated translation suppression of reporter genes, but does not affect siRNA-mediated RNAi.

We next hypothesized that the expression of a specific cellular protein, known to be controlled by miRNAs, might be up-regulated in RCK/p54-depleted cells. Such a protein, human RAS, has been elegantly shown by Slack and colleagues [ 13] to be regulated by the *let*-7 miRNA family. To test our hypothesis, we analyzed RAS protein levels in HeLa cells under two conditions: either *let*-7 function was inhibited by 2′- *O*-Me oligonucleotides complementary to the *let*-7 sequence or RCK/p54 was depleted by RNAi ( Figure 7C). For control experiments, we treated cells with a CDK9 mm siRNA and a 2′ - *O*-Me oligonucleotide complementary to HIV-1 TAR RNA sequence [ 53]. Total cellular protein extracts from equal numbers of cells were prepared and analyzed by immunoblot using monoclonal anti-RAS antibodies directly conjugated with HRP (see Materials and Methods). Actin protein levels were measured as a control using HRP-conjugated anti-actin primary antibody. When cells were treated with *let*-7 inhibitor, RAS protein levels increased, consistent with previous findings [ 13]. Strikingly, RAS protein expression in RCK/p54-depleted cells also showed a robust increase ( Figure 7C) while RAS levels were not significantly affected when cells were treated with control siRNA or 2′- *O*-Me oligonucleotides. RAS mRNA levels did not differ drastically in HeLa cells after treatment with *let*-7 inhibitor, RCK/p54 silencing, or mock treatment ( Figure S3).

To further probe our findings of endogenous RAS regulation by RCK/p54, we co-transfected HeLa cells with RAS 3′ UTR reporter constructs and *let-7* 2′ - *O*-Me inhibitor or siRNA against RCK/p54. For control experiments, we treated cells with a CDK9 mm siRNA and a 2′ - *O*-Me oligonucleotide complementary to HIV-1 TAR RNA sequence [ 53]. Cells transfected with a control siRNA and *let*-7 inhibitor induced more firefly luciferase expression when reporter plasmids contained 3′ UTR sequences for NRAS and KRAS than for the control siRNA and a 2′- *O*-Me oligo control ( Figure 7D), consistent with a recent report [ 13]. Depleting RCK/p54 by siRNA and adding a control 2′- *O*-Me oligo also released translation repression from both NRAS and KRAS vectors. Interestingly, combining these two approaches to release translation suppression, *let-*7 2′- *O*-Me oligo and RCK/p54 depletion, did not show additive effects to induce NRAS and KRAs expression. In all of these experiments, slightly greater firefly luciferase expression was induced from pGL3-NRAS than from KRAS vectors. These results, combined with those of Figure 7A–C, strongly suggest that RCK/p54 is a general translation repressor that associates with RISC to regulate miRNA-mediated translation repression.

## Discussion

We have identified RCK/p54 as a protein that interacts with Ago2 in affinity-purified RISC assemblies to facilitate formation of cytoplasmic P-bodies and that acts as a general translational repressor in human cells. We have shown that depletion of RCK/p54 disrupts P-bodies and disperses the cytoplasmic localization of Ago2. Furthermore, depletion of RCK/p54 did not significantly affect the RNAi function of RISC, although general, miRNA-mediated, and *let*-7-mediated translational repression were released. Together, our results provide significant insights into miRNA mechanisms in human cells (see below).

Several recent reports show that RISC co-localizes with P-bodies, suggesting that they could be the site where RISC degrades target mRNA or represses mRNA translation [ 28– 31]. Isolation of miRNAs on polysomes also links the suppression of protein synthesis with arrest of translation initiation or elongation [ 29]. These studies also demonstrated that human Ago1 and Ago2 co-localize in P-bodies with other cellular proteins, such as Dcp1a, Dcp2, GW182, Lsm1, and Xrn1 [ 28– 31]. A homolog of the P-body protein GW182 in Caenorhabditis elegans is the developmental timing regulator AIN-1, which also interacts with miRISCs and may target argonaute proteins to P-bodies [ 57]. To dissect and understand the relationship between RNAi function and P-bodies, we affinity-purified RISC using Myc-Ago2 and expression vectors of the YFP-tagged P-body proteins, Lsm1, RCK/p54, Dcp2, and eIF4E. Ago2 interacted with these various P-body components in ways that were RNA-dependent or RNA-independent ( Figure 1A). Ago1 and RCK/p54 immunoprecipitated with Ago2 after RNase A treatment of HeLa cell extracts, suggesting that these proteins directly interact with Ago2. Interestingly, RCK/p54, Ago1, and Ago2 were also identified as a component of active RISC programmed with siRNA or miRNA and purified by biotin affinity to streptavidin-conjugated magnetic beads ( Figures 2 and 3). We examined the P-body localization of Ago2 with Lsm1 and RCK/p54 by co-expressing YFP-tagged Lsm1 and RCK/p54 with CFP-Ago2. Interestingly, overexpressing YFP-RCK/p54 in HeLa cells increased the number of P-bodies (from ∼ 8 to ∼ 20 foci/cell). The number of P-bodies containing CFP-Ago2 also increased ( Figure 1B). These results suggested a functional relationship between RCK/p54-Ago interactions and their localization to P-bodies. To visualize protein–protein interactions in vivo, we used FRET as a probe. In cells expressing YFP-RCK/p54 and CFP-Ago2, the FRET efficiency was 21.07% ± 2.52% ( Figure 1C and 1D). We also observed an efficient FRET between YFP-Ago1 and CFP-Ago2; however, FRET between RCK/p54 and Ago1 was moderate (6.41% ± 1.96%). Since FRET is quite sensitive to the orientation of the donor: acceptor pair, it is possible that CFP and YFP in Ago1 and RCK/p54 are not suitably positioned for efficient energy transfer. Nonetheless, the FRET efficiency between Ago1 and RCK/p54 was significantly above the 0.9% background. Taken together, these results demonstrate that Ago1, Ago2, and RCK/p54 directly interact in vivo.

Where in the cell does this physical interaction between RISC and RCK/p54 take place and does it require P-body structures? To address these questions, we disrupted P-bodies in cells by depleting Lsm1 and immunopurified endogenous miRISC, and analyzed its ability to cleave a target mRNA with perfect complementarity to *let*-7 miRNA ( Figure 5). We observed no significant difference in miRISC activities purified from Lsm1-depleted and non-depleted cells ( Figures 3 and 5) even though the P-body structures were disrupted and the total number of P-bodies per cell had significantly decreased. These results show that RCK/p54 association with RISC does not require P-bodies and suggest that localization of RISC to P-bodies is the outcome of translation repression by miRISC.

Having established the physical interactions of RCK/p54 with RISC, we questioned the functional relevance of these interactions in the RNAi pathway and how they could facilitate RISC activity. To that end, we depleted RCK/p54 in HeLa cells and analyzed RNAi activities in vivo and in vitro ( Figure 6). Depletion of RCK/p54 clearly disrupted the P-body structures and dispersed Ago2 localization throughout the cytoplasm ( Figure 4). Surprisingly, depleting RCK/p54 did not significantly affect siRNA-mediated gene silencing. Similar results showing the lack of P-body involvement in RNAi have been reported in *Drosophila* [ 39]. In that system, depletion of the P-body proteins, GW182 or Dcp1:Dcp2 decapping complex, showed that these proteins were required for miRNA-mediated gene silencing *,* but RNAi efficiency was not affected, suggesting that these two gene-silencing mechanisms were independent in *Drosophila* [ 39]. In addition, two recent studies reported that mammalian GW182 interacts with Ago2 and plays a role in miRNA function [ 40] and in siRNA-mediated RNAi [ 41]. Interestingly, depleting GW182 disrupted cytoplasmic foci and interfered with RNAi activity [ 41]. It is possible that GW182 is directly involved in human RISC function and does not spatially require P-bodies for its function. As an alternative approach to disrupting P-bodies, we treated HeLa cells with the translation inhibitor, cycloheximide, which did not significantly affect RNAi activities in vivo and in vitro (unpublished data). Taken together, these results strongly suggest that functional siRISC is assembled before locating to P-bodies, and P-bodies are not prerequisite sites for siRNA-mediated gene silencing.

To determine the role of RCK/p54 in miRNA-mediated translation repression, we used a CXCR4 reporter system [ 56], in which siRNA- or miRNA-reporter constructs harbor 1 × perfectly matched or 4 × bulged CXCR4 siRNA target sites, respectively in the 3′ UTR of RL mRNA [ 56]. In this system, perfectly matched sequences are cleaved via siRISC and bulge-containing sequences are targets for translation suppression by miRISC. We observed that depletion of RCK/p54 released only miRNA-mediated gene suppression and had no effect on siRNA-mediated gene silencing ( Figure 7B). Depletion of GW182, an argonaute-interacting P-body protein, released gene suppression by miRNA and moderately affected reporter constructs containing perfectly matched target sequences, consistent with previous findings [ 39– 41]. Ago2 depletion significantly released siRNA-mediated gene silencing and moderately affected reporters containing 4 × bulged sequences. Knockdown of Lsm1 had no significant effect on either miRNA- or siRNA-mediated gene silencing. These results show that RCK/p54 depletion releases miRNA-mediated translation suppression of reporter genes and that this function of RCK/p54 was not significantly affected when P-bodies were disrupted by depleting Lsm1.

To test our hypothesis that RCK/p54 mediates the translational repression of endogenous miRNA targets in P-bodies, we examined RAS protein levels in RCK/p54-depleted cells. We chose RAS because it is an endogenous target of *let*-7 *,* and 3′ UTRs of human *RAS* genes contain multiple complementary sites for *let*-7 to bind and regulate RAS expression levels [ 13]. Furthermore, *let*-7 inhibitors are known to enhance RAS protein expression in HeLa cells [ 13]. Depleting RCK/p54 in HeLa cells up-regulated RAS protein, and this increase in RAS levels was higher than that in general translation of control actin ( Figure 7C), suggesting that multiple sites of *let*-7 miRISC binding to target 3′ UTR dictate the potency and specificity of translation suppression.

RCK/p54, the human homolog of yeast Dhh1p, is a member of the ATP-dependent DEAD box helicase family and was originally identified as a proto-oncogene [ 42]. In human cells, RCK/p54 interacts in P-bodies with the translation initiation factor, eIF4E [ 33]. The *Xenopus* homolog of RCK/p54, Xp54, which interacts with eIF4E and forms RNA-dependent oligomers, represses the translation of mRNA in oocytes and eggs [ 43]. In yeast, Dhh1p interacts with the decapping and deadenylase complex and functions in translational repression [ 44]. Dhh1p has recently been shown to stimulate translational repression by inhibiting production of the pre-initiation complex [ 45]. Dhh1p and RCK/p54 also inhibit the mRNA translation driven by internal ribosomal entry sites in vitro, suggesting that this protein is a general translational repressor that may not require a cap structure for its function [ 45]. However, the in vivo function of Dhh1p required translational initiation [ 45]. Translation repression by miRISC and P-body localization require the 5′-cap structure in the target mRNA [ 29, 58], which provides a unique and elegant control mechanism for translation and its regulation (reviewed in [ 59, 60]). Recently, Petersen et al. reported an intriguing study showing that miRNA represses translation after initiation by a ribosome drop-off mechanism [ 61]. Therefore, it is possible that RCK/p54 interacts with miRISC and blocks translation elongation by binding to its multiple target sites with high affinity and creates a barrier for elongating ribosomes (see below).

**Figure 8:**
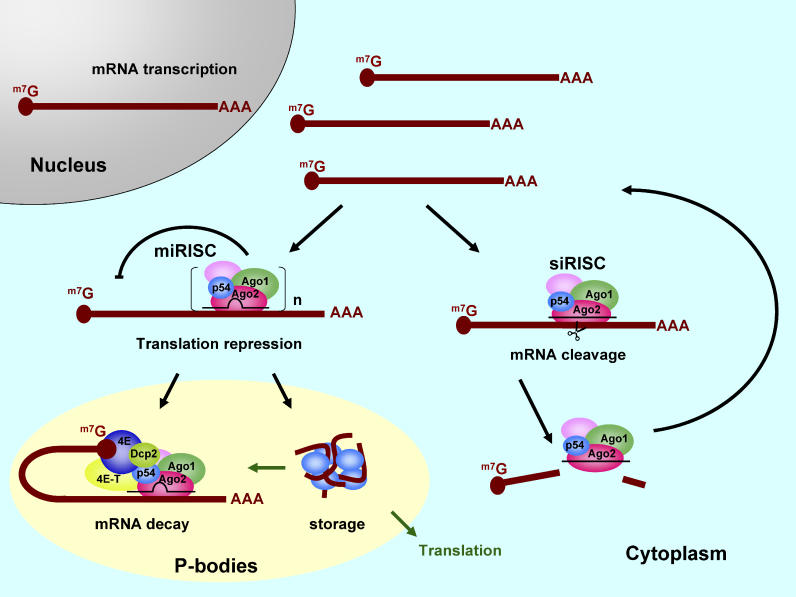
A Model for Human RISC Function Involving miRNA and siRNA RISC contains Ago2 (red), Ago1 (green), RCK/p54 (blue, labeled p54), and other known (e.g., Dicer and TRBP) and unidentified proteins (pink) and is distributed throughout the cytoplasm. siRISC binds to its target mRNA by perfectly matching base pairs, cleaves the target mRNA for degradation, recycles the complex, and does not require P-body structures for its function. Multiple numbers *(n)* of miRISC bind to target mRNA by forming a bulge sequence in the middle that is not suitable for RNA cleavage, accumulate in P-bodies, and repress translation by exploiting global translational suppressors such as RCK/p54. The translationally repressed mRNA is either stored in P-bodies or enters the mRNA decay pathway for destruction. Depending upon cellular conditions and stimuli, stored mRNA can either re-enter the translation or mRNA decay pathways.

We therefore propose that the function of RISC assemblies in cells can be described by a model with two independent pathways, depending on whether RNAi is programmed by the guide strand of siRNA or miRNA. In the first pathway, the RISC recognizes a perfectly matched target mRNA and functions as siRISC ( Figure 8) by cleaving its target mRNA. This target mRNA cleavage activity and rapid turnover of siRISC induce the subsequent target mRNA degradation by exonucleases and does not require P-bodies. In the second pathway, the RISC recognizes the imperfectly matched target and functions as miRISC ( Figure 8). Multiple copies of miRISC containing RCK/p54 could initiate an oligomerization event that sequesters the whole RNP and transports it to the P-bodies. This sequestration could also explain the cooperative effects of RISC function that enhance translation repression [ 56]. Once in P-bodies, translationally repressed mRNA could stay in oligomeric structures for storage or it could form a complex with decapping enzymes and cap binding proteins that would lead to the mRNA decay pathway. In other words, the miRNA in RISC could provide the sequence specificity and RCK/p54 could be the effector molecule that shuttles the target mRNA toward the fate of storage or processing in P-bodies. Since P-bodies are not a prerequisite for RCK/p54 to assemble into RISC or to function, it is possible that miRISC blocks protein synthesis by enhancing the ribosome drop-off during elongation of translation [ 61]. We propose that miRISC localization to P-bodies is the outcome of translation repression and not a prerequisite for miRISC function. Stored mRNA in P-bodies could reenter the active translation or mRNA decay pathway depending upon cellular conditions or stimuli that are presently unknown. How long mRNA translation can be repressed in the cytoplasm and which factors trigger mRNA transport to P-bodies are exciting areas for future research.

Our results on the interactions between RCK/p54 and the RNAi machinery suggest an intriguing role for miRNA function in development and carcinogenesis. However, most targets of miRNA have not yet been identified. A growing body of evidence suggests that miRNAs are important in human disease, including cancers [ 9– 12]. For example, relatively low levels of *let*-7 miRNA up-regulate RAS protein in lung cancer cells, demonstrating a possible role of miRNA in tumorigenesis [ 13]. Two homologs of RCK/p54, Xp54 in *Xenopus* and Me31b in *Drosophila,* control the translation of maternal mRNA in oocytes [ 43, 62, 63]. Altered regulation of RCK/p54 expression levels has been implicated in the development of human colorectal tumors [ 64, 65] and in hepatitis C virus-related chronic hepatitis [ 66]. Overexpression of RCK/p54 and Dhh1p increases the number of Ago2-containing P-bodies ( Figure 1B; [ 33, 45]), suggesting that RCK/p54 is a critical factor in controlling cellular translation. Our results have identified RCK/p54 as an essential component of miRISC-mediated translation repression and provide a functional link between miRISC and P-bodies. In the context of spatially and temporally restricted miRNA expression, this system allows for exquisite regulation of protein expression by target mRNA. Therefore, perturbations of either miRNA or RCK/p54 expression levels can have deleterious consequences for the cell. What determines the balance between active translation and repression of mRNAs targeted by miRISC, and how cells control the specificity of this repression are key directions for future investigation.

## Materials and Methods

### Expression vectors.

Ago1 and Ago2 expression vectors with N-terminal YFP- or CFP-epitope tags were generated by PCR amplification of *Ago*1 and *Ago*2 coding sequences from pMyc-Ago1 and pMyc-Ago2 followed by cloning into the *Xba*I and *EcoR*I sites of pEYFP-C1 and pECFP-C1 (BD Biosciences, Palo Alto, California, United States). Expression vectors were kindly provided by four laboratories: pMyc-Ago1/2 (Dr. G. Hannon); pFlag-Ago1/Ago2 (Dr. T. Tuschl); pGL3-control, pGL3-KRAS, and pGL3-NRAS (Dr. F. Slack); and pRL-TK-1P and pRL-TK-4B (Dr. P. Sharp).

Vectors for expressing YFP-tagged Lsm1, RCK/p54, eIF4E, and Dcp2 were generated through PCR amplification of their coding sequences from 293T cDNA followed by cloning into the *BglII* and *SalI* sites of pEYFP-C1 [ 67].

### siRNAs.

siRNAs against GFP, human Ago2, RCK/p54, Lsm1, and CDK9 mismatch were synthesized by Dharmacon (Dharmacon, Lafayette, Colorado, United States) and 2′-OH deprotected according to the manufacturer's protocols. The sequences of siRNAs (passenger strand) for our experiments were:

GFP: 5′- GCAGCACGACUUCUU CAAGdTdT-3′; hAgo2: 5′- GCACGGAAGUCCAUCUGAAdTdT-3′

RCK/p54: 5′- GCAGAAACCCUAU GAGAUUUU-3′; CDK9mm: 5′- CCAAAGCUCU CCCCUAUAAdTdT-3′; Lsm1: 5′-GU GACAUCCU GCCACCUCACUU-3′

### Cell culture and transfection.

HeLa cells were cultured in Dulbecco's minimal essential medium (DMEM) with 10% fetal bovine serum (FBS) at 37 °C with 5% CO _2_. Cells were transfected using Lipofectamine (Invitrogen, Carlsbad, California, United States) according to the manufacturer's protocol.

### Immunoprecipitation and immunoblotting.

TCEs were prepared by incubating cells in lysis buffer (20 mM HEPES [pH 7.9], 10 mM NaCl, 1 mM MgCl _2_, 0.2 mM EDTA, 0.35% [v/v] Triton X-100, 1/100 [v/v] dilution in protease inhibitor cocktail) and centrifuging at 14,000 rpm for 10 min at 4 °C. Protein concentration was determined by Dc protein assay (Bio-Rad, Hercules, California, United States). To examine the RNA dependence of protein–protein interactions, TCEs (250 μg) were treated before immunoprecipitation with 0.2 μg/ul of RNase A for 20 min at room temperature. Myc-tagged proteins were precipitated by incubating overnight with polyclonal rabbit anti-Myc antibodies directly conjugated to agarose beads (Santa Cruz Biotech, California, United States). Samples were washed four times in lysis buffer and eluted by boiling for 5 min at 100 °C in SDS-PAGE sample loading buffer, separated by SDS-PAGE, and analyzed by immunoblot. For immunoblotting, antibodies included monoclonal mouse anti-GFP (BD Biosciences), anti-eIF4E and anti-Myc (Santa Cruz Biotech), anti-Flag (Sigma, St. Louis, Missouri, United States); polyclonal rabbit anti-Myc (Santa Cruz Biotech), anti-DDX6 (rck/p54; Bethyl Laboratories, Montgomery, Texas, United States); and polyclonal chicken anti-Lsm1 (GenWay Biotech Incorporated, San Diego, California, United States).

To assess the ability of endogenous immunopurified miRISC to cleave target mRNA with perfect complementarity to *let*-7 miRNA, in vitro cleavage assays for *let*-7 miRISC were conducted. Aliquots (30 μl) of magnetic protein A beads (Dynal, Norway) were preincubated with rabbit IgG, anti-Ago2, or anti-RCK/p54 antibodies according to the manufacturer's protocol and incubated overnight at 4 °C with 500 μg HeLa cytoplasmic extracts. Beads were then washed three times with Na-phosphate buffer [pH 8.0], one time with lysis buffer, and resuspended in 30 μl of lysis buffer. Aliquots (10 μl) of these beads were used in each *let*-7 miRISC cleavage assay.

### Live cell imaging, FRET, and immunofluorescence.

HeLa cells were cultured in 35 mm dishes with glass coverslip bottoms (MatTek Corporation, Ashland, Massachusetts, United States). Expression vectors for CFP- or YFP-tagged proteins were transfected into cells using Lipofectamine as described above. 24 h later, the live cells were monitored for CFP and YFP signals of the transiently expressed proteins. The signals were detected by a Leica (Wetzlar, Germany) confocal imaging spectrophotometer system (TCS-SP2) attached to a Leica DMIRE inverted fluorescence microscope equipped with an argon laser, two HeNe lasers, an acousto-optic tunable filter (AOTF) to attenuate individual visible laser lines, and a tunable acousto-optical beam splitter (AOBS). A 63 × 1.32 NA oil-immersion objective was employed. For FRET studies, HeLa cells co-expressing CFP- and YFP-tagged proteins were fixed with 4% paraformaldehyde in PBS at room temperature for 20 min and washed three times with PBS. After the final wash, cells were visualized with a Leica confocal imaging system as described above. FRET experiments were performed by an acceptor photobleaching method as described [ 48, 49, 68]. FRET efficiencies were measured and images were analyzed using Leica confocal software. For immunofluorescence studies, cells transfected with Myc-Ago2 were fixed with 4% paraformaldehyde in PBS at room temperature for 20 min and permeabilized with 0.25% (v/v) Triton X-100 for 5 min. Samples were washed three times with PBST (0.1% [v/v] Triton X-100 in PBS) and blocked for 30 min in PBST containing 2% (w/v) BSA. Primary and secondary antibodies were diluted in blocking solution during incubation. Secondary antibodies against rabbit IgG, mouse IgG, and chicken IgY were directly conjugated to Alexa Fluor dyes (Molecular Probes, Eugene, Oregon, United States). After the final wash, samples were counterstained with Hoechst 33258 to visualize nuclei with a Leica confocal imaging system as described above.

### In vitro cleavage assay for siRISC and miRISC.

HeLa cells sequentially transfected with siRNAs against P-body components and EGFP were harvested 24 h after the second transfection. Cytoplasmic extracts of HeLa cells were prepared as described above and the protein concentration was determined by Dc protein assay (Bio-Rad). Target mRNAs were prepared and in vitro cleavage by GFP-siRISC and *let*-7-miRISC was assayed as described [ 53].

### Dual fluorescence assay.

A dual fluorescence assay was used to quantify the RNAi activity of siRNAs against GFP. To quantify RNAi effects, cell lysates were prepared from siRNA-treated cells 24 h post-transfection. Total cell lysate (150 μg in 200 μl of reporter lysis buffer) was measured using a Safire plate reader (TECAN). GFP fluorescence was detected in cell lysates by exciting at 488 nm and recording emissions from 504–514 nm. The spectrum peak at 509 nm represents the fluorescence intensity of GFP. RFP fluorescence was detected in the same cell lysates by exciting at 558 nm and recording emissions from 578–588 nm. The spectrum peak at 583 nm represents the fluorescence intensity of RFP. The fluorescence intensity ratio of target (GFP) to control (RFP) fluorophores was determined in the presence of siRNA duplexes and normalized to the emissions measured in mock-treated cells. Normalized ratios < 1.0 indicated specific RNA interference.

### Incorporation of [ ^35^S]methionine.

HeLa cells cultured in 6-well plates were transfected with siRNAs against RCK/p54 or CDK9 mismatch as a scramble control. 24 h post-transfection, cells were incubated for 1 h in culture medium lacking methionine and cysteine, and metabolically labeled by incubating in culture medium containing 100 μCi/ml Tran ^35^S-label (MP Biomedicals, Irvine, California, United States). At 0, 15, 30, 60, and 120 min after metabolic labeling, cells were washed twice and harvested in 300 μl M-PER buffer (Pierce, Rockford, Illinois, United States) with protease inhibitors (Sigma). For each experiment, two extra sets of siRNA-treated cells were trypsinized and counted for total cell numbers. Cell lysates (50 μl) were incubated with 10 μl of 100% TCA on ice for 30 min, and the protein precipitate was collected on GF/C filter paper (Whatman, Clifton, New Jersey, United States), washed with 5 ml of 95% ethanol and counted in scintillation fluid.

### Dual luciferase assay for *let-*7 miRNA-mediated gene silencing.

A dual luciferase assay was used to quantify the effects of *let*-7- and RCK/p54-mediated gene silencing on human NRAS or KRAS 3′ UTR. HeLa cells cultured in 6-well plates were co-transfected with 0.8 μg/well *Pp*-luc-expressing vectors (pGL3-control, pGL3-NRAS, or pGL3-KRAS) and with 100 nM *let-7*-2′- *O*-Me inhibitor or 50 nM siRNA against RCK/p54. In all experiments, transfection efficiencies were normalized to those of cells co-transfected with the *Rr*-luc-expressing vector (pRL-TK; 0.1 μg/well). TCEs were prepared 48 h post-transfection and dual luciferase assays (Promega, Madison, Wisconsin, United States) were performed according to the manufacturer's protocol and quantified with a Victor2 multilabel counter (Perkin Elmer). The *Pp*-luc/ *Rr*-luc signals were normalized to those from pGL3-control-transfected cells, showing *let-7*-regulated gene silencing of RAS 3′ UTR.

### siRNA or miRNA luciferase reporter assays.

To analyze siRNA- or miRNA-mediated gene-silencing events, we chose the CXCR4 mRNA reporter system, in which siRNA- or miRNA-reporter constructs harbor 1 × perfectly matched or 4 × bulged CXCR4 siRNA target sites, respectively, in the 3′ UTR of RL mRNA [ 56]. HeLa cells transfected with siRNAs directed against P-body proteins (RCK/p54, GW182, Lsm1, Ago2) were co-transfected again with siRNA or miRNA reporters in the absence or presence of 25 nM CXCR4 siRNA. At 24 h post-transfection, cells were harvested and RL activities were analyzed. RL signals were normalized to the FL signals from cells co-transfected with pGL3 plasmid as control.

## Supporting Information

Figure S1 Subcellular Localization of Endogenous Ago2 in HeLa Cells HeLa cells were analyzed by immunofluorescence using antibodies against endogenous Ago2 (A) and Lsm1 (B), and stained with Hoechest 33258 to visualize the nucleus. The images were digitally merged to indicate co-localization of Ago2 and Lsm1 (C). Arrows point to P-bodies.
(4.2 MB TIF)

Figure S2 Specific Depletion of RCK/p54 in HeLa Cells by RNAi (A) Specific knockdown of RCK/p54 in HeLa cells. HeLa cells were transfected with 50 nM siRNA against RCK/p54, harvested at 24, 48, and 72 h post-transfection, and TCEs were prepared. Analysis by immunoblot shows the specific knockdown of RCK/p54 protein without changing the protein levels of Lsm1 or Ago2.
(B) Specific depletion of RCK/p54 mRNA after siRNA treatment. Total RNA samples (3 μg) from HeLa cells transfected with siRNA against RCK/p54 were reverse-transcribed and analyzed by quantitative PCR to quantify mRNA levels. RCK/p54 mRNA levels were normalized to GAPDH mRNA and are presented relative to mock treatment. Data are from two representative, independent experiments.
(1.7 MB TIF)

Figure S3 *Let*-7 Inhibition Does Not Affect RAS mRNA Levels
Total RNA samples (3 μg) from HeLa cells transfected with 100 nM of *let*-7 2′- *O-*Me oligonucleotides or 50 nM siRNA against RCK/p54 were reverse-transcribed and analyzed by quantitative PCR to quantify mRNA levels. RAS mRNA levels were normalized to GAPDH mRNA and are presented relative to mock treatment. Data are from two representative, independent experiments.
(1.5 MB TIF)

### Accession Numbers

The RefSeq ( http://www.ncbi.nlm.nih.gov/RefSeq) accession numbers for the proteins discussed in this paper are Ago1 (NM_012199), Ago2 (NM_012154), Dcp2 (NM_152624), eIF4E (NM_001968), Lsm1 (NM_014462), and RCK/p54 (NM_004397).

## Abbreviations

2′ - *O*-Me: 2′ - *O*-Methyl
Ago1: Argonaute1
Ago2: Argonaute2
CFP: cyan fluorescent protein
EGFP: enhanced GFP
FRET: fluorescence resonance energy transfer
GFP: green fluorescent protein
GL: *Photinus pyralis* luciferase
miRISC: microRNA-induced silencing complex
miRNA: microRNA
nt: nucleotide
P-body: mRNA processing body
RISC: RNA-induced silencing complex
RL: *Renilla reniformis* luciferase
RNAi: RNA interference
RFP: red fluorescent protein
RNP: ribonucleoprotein
siGFP: siRNA targeting GFP
siRISC: RISC programmed by siRNA
siRNA: short interfering RNA
TCE: total cell extract
YFP: yellow fluorescent protein
